# Successful deslorelin implant treatment in a neutered female Pomeranian dog with Alopecia X

**DOI:** 10.29374/2527-2179.bjvm001325

**Published:** 2025-04-22

**Authors:** Priscila Alves Bastos Coimbra, Aline Bomfim Vieira

**Affiliations:** 1 Priscila Dermatovet Consultations, Rio de Janeiro, RJ, Brazil.; 2 Biomedical Sciences Department, Cornell University College of Veterinary Medicine, Ithaca, NY, United States.

**Keywords:** GnRH analog, suprelorin, hair cycle arrest, noninflammatory alopecia, sex steroids, Análogo do GnRH, suprelorin, parada do ciclo capilar, alopecia não inflamatória, esteroides sexuais

## Abstract

The Pomeranian dog is recognized as one of the predisposed breeds for alopecia X, a noninflammatory hair cycle arrest disorder. Although it is considered a cosmetic issue unrelated to systemic illness, having a pet with alopecia can be distressing for owners. Sex steroid imbalance seems to influence the development of alopecia X, but the exact pathogenesis is still unknown. Although some treatment alternatives are currently available for alopecia X, results can vary, as well as costs, risks, and owner adherence. GnRH analogs have been used extensively in the modulation of sex hormone synthesis in humans. Previous studies support the use of the GnRH analog in treating male dogs with alopecia X, but current literature discourages its use in females. We aim to report the first scientific description of a neutered female dog with alopecia X successfully treated with a 4.7 mg deslorelin acetate implant after failing to respond to melatonin. Profuse hair regrowth took 9 months to occur after the first implant, but the treatment was considered uncomplicated, cost-effective, and safe.

## Resumo

O Lulu da Pomerânia (Spitz Alemão Miniatura) é reconhecido como uma das raças predispostas à alopecia X, uma alopecia não inflamatória que interrompe o ciclo capilar. Embora seja considerado um problema cosmético não relacionado à doenças sistêmicas, ter um animal de estimação com alopecia pode ser angustiante para os donos. O desequilíbrio dos esteroides sexuais parece influenciar o desenvolvimento da alopecia X, mas a patogênese exata ainda é desconhecida. Embora algumas alternativas de tratamento estejam atualmente disponíveis para a alopecia X, os resultados podem variar, assim como os custos, os riscos e a adesão do proprietário. Os análogos do GnRH têm sido amplamente utilizados na modulação da síntese de hormônios sexuais em humanos. Estudos anteriores suportam o uso do análogo do GnRH no tratamento de cães machos com alopecia X, mas a literatura atual desencoraja o uso em fêmeas. Nosso objetivo é relatar a primeira descrição científica de uma cadela castrada com alopecia X tratada com sucesso usando implante de acetato de deslorelina 4,7 mg após não responder ao tratamento com melatonina. O crescimento profuso do pêlo demorou 9 meses para ocorrer após o primeiro implante, mas o tratamento foi considerado descomplicado, econômico e seguro.

## Introduction

A healthy hair cycle maintenance depends on numerous factors, such as follicular stem cells, several molecules derived from epithelial, mesenchymal, and neuroectodermal cells, and the external matrix of the follicular and dermal environment. It can also be influenced by factors like hormones, age, genetics, and time of the year (Chen & Chuong, 2012). Once this highly conserved and tightly regulated process is disturbed, alopecia can develop. Primary noninflammatory alopecia is caused by a decreased formation of cytodifferentiation of hair follicles or hair shafts (Welle, 2023). Some breeds, like poodles and the Nordic and ‘plush-coated’ breeds, are born with an intact hair coat but may develop noninflammatory alopecia early in life (Frank, 2005; Müntener et al., 2012).

The Pomeranian dog is recognized as one of the predisposed breeds for alopecia X, a noninflammatory hair cycle arrest disorder (Frank, 2005; Mausberg et al., 2007; Müntener et al., 2012; van Hensbergen et al., 2025). This disorder affects male and female young adult dogs independently of their neuter status and has several other names in the past, including adult-onset hyposomatotropism, growth hormone-responsive alopecia, pseudo-Cushing's disease, castration-responsive alopecia, adrenal hyperplasia-like-syndrome. The multiple nomenclature reflects not only the unknown pathogenesis but also the likely hormonal imbalance that is behind its development (Cerundolo et al., 2007; Frank, 2005, 2006; Frank et al., 2003, 2004).

Clinical signs of Alopecia X consist of partial to complete alopecia of the neck, tail, caudal-dorsum, perineum, caudal thighs, and ultimately trunk, sparing the head and forelimbs. In addition, the skin may become hyperpigmented, primarily in areas of alopecia (Frank et al., 2004; Welle, 2023). The diagnosis is usually made by ruling out other causes of noninflammatory alopecia, especially hypothyroidism, and hypercortisolism, as well as combining clinical signs with dermatological exams (Müntener et al., 2012; Welle, 2023; Zanna et al., 2024).

Different therapies, including melatonin, trilostane, mitotane, microneedling, laser therapy, and castration, have been suggested with variable results (Amado Martins et al., 2024; Cerundolo et al., 2004; Frank et al., 2004; Huang et al., 2009; Kang et al., 2024; Stoll et al., 2015). GnRH is a hypothalamic neuronal decapeptide hormone that controls the hypothalamic-pituitary-gonadal axis, which is crucial for regulating reproduction (Casteel & Singh, 2020). It binds to receptors in the pituitary gland to stimulate the release of the follicular stimulating hormone (FSH) and luteinizing hormone (LH), also known as gonadotrophins, which, in turn, stimulate the production and release of testosterone by the male testes and estrogen by the female ovaries and placenta (Wu et al., 2021). Deslorelin acetate is a long-acting gonadotrophin-releasing hormone (GnRH) analog used to suppress fertility in male dogs, male cats, and prepubertal female dogs (Amaral et al., 2023; Gontier et al., 2022; Lucas, 2014). It is available as a subcutaneous implant that will release a slow and continuous hormone dose, suppressing the reproductive endocrine system by preventing the production of gonadotrophins FSH and LH. In the last 12 years, two studies and one case report have shown that deslorelin acetate can promote hair regrowth in male dogs with alopecia X (Albanese et al., 2014; Cerundolo & Warren, 2013; Layne & Richmond, 2018), but its use in neutered female dogs is currently discouraged (Albanese et al., 2014). We aim to report the first successful treatment of a neutered female Pomeranian dog with alopecia X using a 4,7 mg deslorelin acetate implant, after failing to respond to melatonin.

## Case description

**Figure 1:**
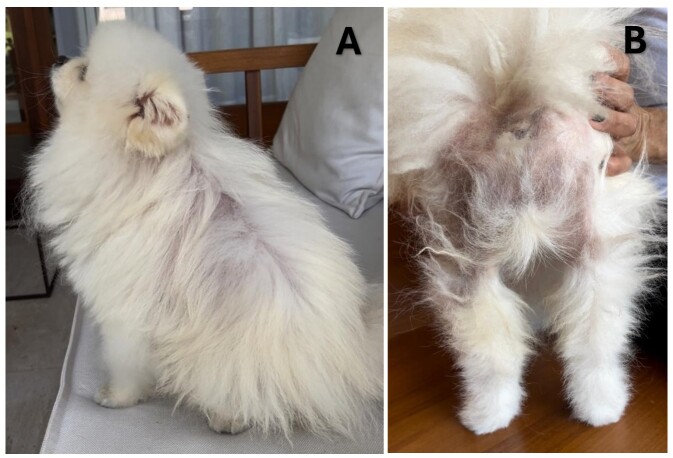
A 2-year-old neutered female Pomeranian dog with alopecia X shows alopecia and hyperpigmentation (A) in the trunk and (B) peri vulvar and caudal thighs areas.

**Figure 2:**
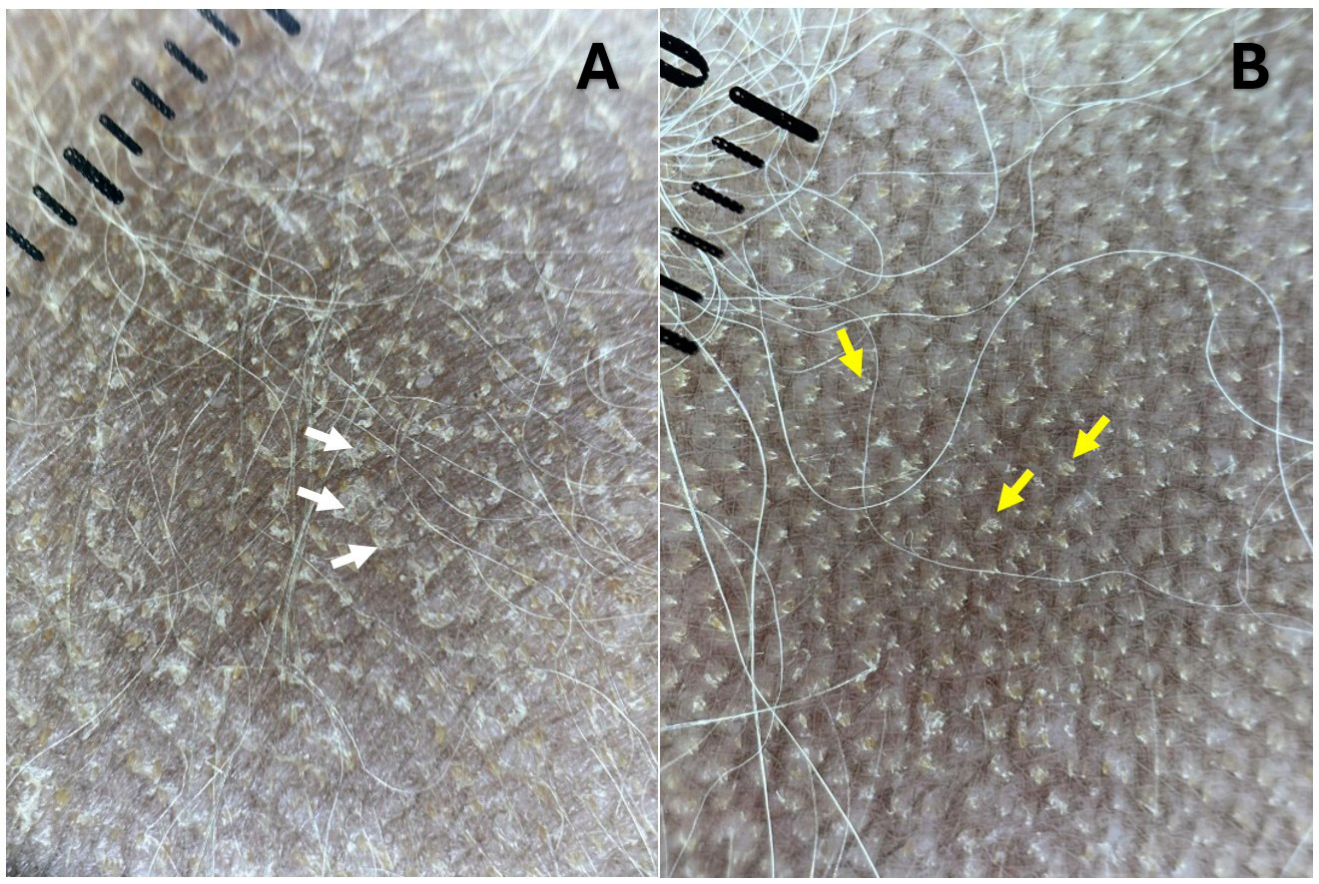
Dermatoscopy images of the affected skin of a 2-year-old neutered female Pomeranian dog with alopecia X. Images A and B show an amplified view of the alopecia and hyperpigmentation trunk areas. (A) scale over the skin (white arrows), (B) short hairs (yellow arrows) mixed with amorphous keratoseborrheic-like material (follicular plugging).

A 2-year-old female neutered Pomeranian dog (3.5 Kg) imported from South Korea and living in Rio de Janeiro, Brazil, since 6 months old was evaluated for a history of progressive hair loss during the last year. Pruritus was never present. During the physical examination, the dog was otherwise healthy except for the bilaterally symmetric noninflammatory alopecia localized in the skin's cervical, trunk (Figure 1A), perineal, and caudal thigh areas (Figure 1B). Both primary and secondary hair were absent in some areas, while others, like the perineum and caudal thighs, kept only some secondary hair with a wool-like appearance. The skin in alopecic areas was dry, with a generalized scale over the pigmented areas (Figure 2A), and under dermatoscopy, short hairs were mixed with amorphous keratoseborrheic-like material (follicular plugging) (Figure 2B). Hyperpigmentation was mild in the cervical region and trunk (Figure 1A) and moderate to severe in the caudal thighs (Figure 1B). The skin and hair of the head and forelimbs were not affected.

The diagnostic plan was initiated with skin impression cytology and deep skin scrapings. No inflammatory cells or microorganisms were identified in the cytology. Deep scrapings ruled out Demodex mites. Other diagnostic tests were performed, including complete blood count, biochemical profile, ACTH stimulation test with measurement of cortisol, and 17-OH-progesterone, serum free thyroxine (fT4 - by equilibrium dialysis), thyroid stimulating hormone (TSH), and an ultrasound. Although a punch biopsy was suggested, it couldn’t be performed. All results were within normal reference ranges or negative. Based on the history and signalment, clinical, and laboratory findings, the most likely diagnosis was Alopecia X (hair cycle arrest disorder).

**Figure 3:**
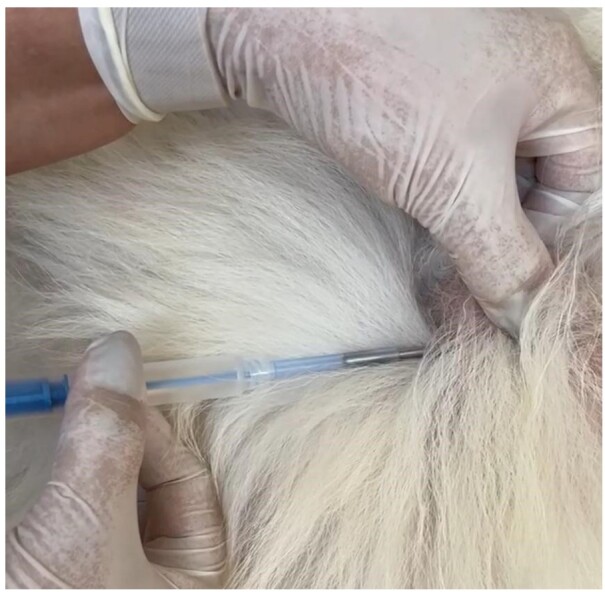
2-year-old neutered female Pomeranian dog with alopecia X receiving a subcutaneous application of the deslorelin acetate implant, a GnRH inhibitor, in the interscapular region.

After discussing treatment options, hydration of the affected skin (CeraVe® daily moisturizing lotion, CeraVe LLC, NY, USA) and compounded oral melatonin (3mg/ BID) were initiated and maintained for 3 months. The dose was then doubled (6 mg/ BID) for 3 more months. Although the skin hydration improved, decreasing follicular plugging under dermatoscopy, no success in hair regrowth occurred, and melatonin was suspended. Then, a 4.7 mg deslorelin implant (Suprelorin®, Virbac, Peptech Animal Health/Virbac, NSW, Australia) was injected subcutaneously into the interscapular space (Figure 3). The dog was rechecked initially 2, 6, and 9 months after the first implant.

**Figure 4:**
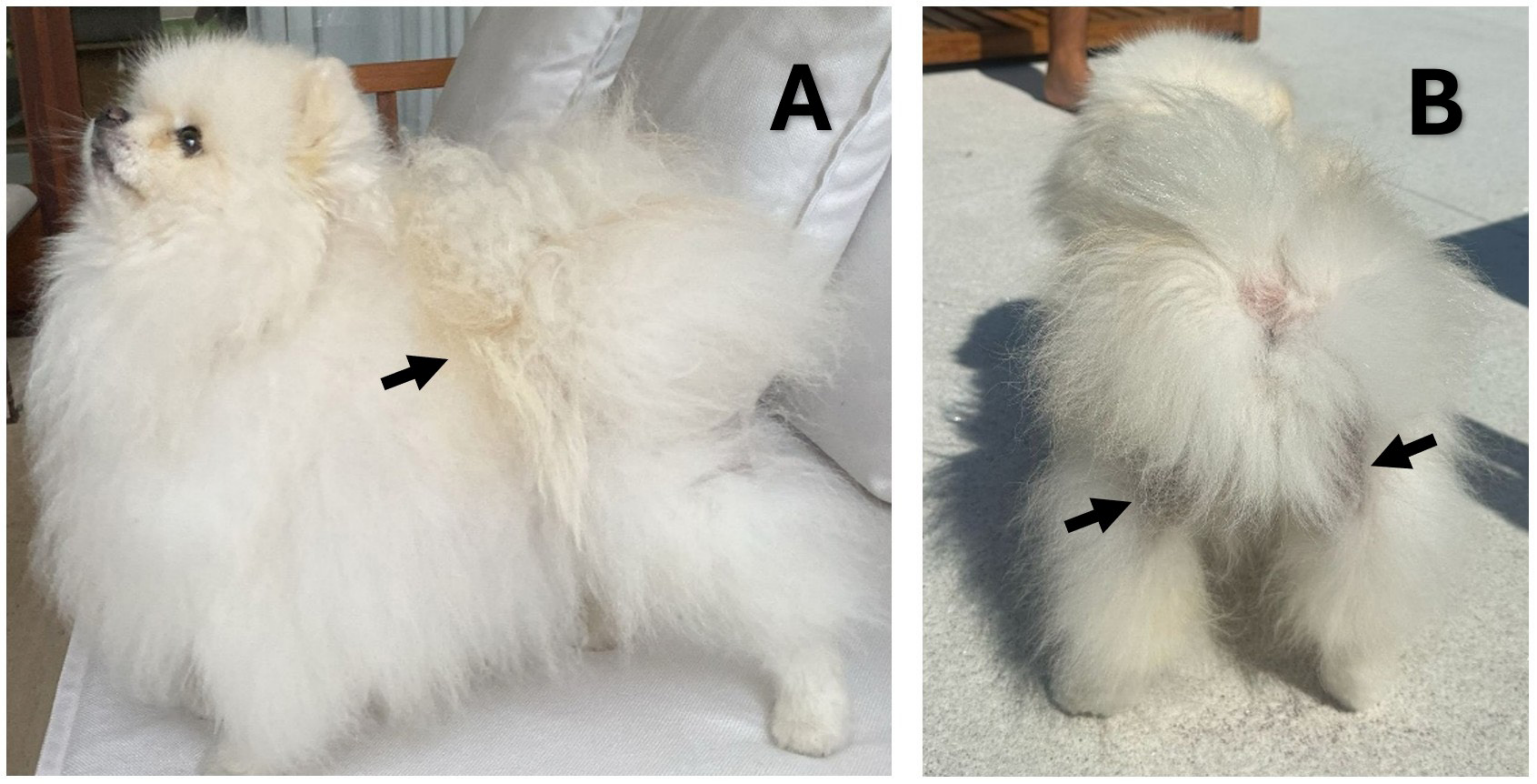
2-year-old neutered female Pomeranian dog with alopecia X, after eleven months of treatment with a 4.7 mg deslorelin implant, a GnRH inhibitor. (A) Note the profuse hair re-growth in the trunk. Some new hairs have a softer appearance and darker color (arrow) compared to the original hair. (B) There is still some alopecia and hyperpigmentation in the caudal thigh area (arrows).

After 60 days, only a discrete response could be seen, with little hair regrowth in the trunk, cervical, and perineal areas. After 180 days, there was a mild regrowth of hair in the same areas. Finally, after 270 days, the female dog started a profuse hair regrowth in all previously affected areas. Since the area of the perineum and caudal thighs were still not fully recovered, a new implant was then injected. Two months after the second implant (eleven months after the beginning of the treatment), the animal has fully recovered the cervical and trunk areas (Figure 4A). Some of the new hairs in the trunk area have a softer appearance and a darker color compared to the original hair (Figure 4A). There is still some alopecia and hyperpigmentation in the caudal thigh area (Figure 4B), but the owner is very satisfied. Overall, the skin is now healthy and well-hydrated. The owner reported no adverse effects.

## Discussion

Alopecia X is a common dermatologic problem with unknown pathogenesis and sometimes frustrating treatment results for veterinarians and owners. Although it is considered a cosmetic issue unrelated to systemic illness, having a pet with alopecia can be distressing for owners. Our report is the first one to contest the current knowledge and show that females with alopecia X can also be successfully treated with a deslorelin implant. Furthermore, we show that profuse hair regrowth could take 9 months to occur after 4.7mg of deslorelin acetate, which is more than the expected 6-month reported duration of the implant. Finally, our case supports using the deslorelin implant as an uncomplicated, cost-effective, and safe treatment alternative for alopecia X in dogs, regardless of gender, including the ones not responsive to melatonin.

In physiological conditions, GnRH is produced by the hypothalamus in a pulsatile manner, and its synthesis is regulated by circulating testosterone levels in males and estrogens in females (Casteel & Singh, 2020). Treatment with GnRH agonists produces an initial transient increase in sex hormones, but with continued non-pulsatile stimulation, LH and FSH synthesis are inhibited, and estrogen and testosterone levels decline (Wu et al., 2021). Since the female dog presented here was neutered at 6 months old, the mechanism of action leading to successful hair regrowth after the implant is probably related to factors other than gonadal sexual hormone production and could include adrenal sex steroid production and sex hormone metabolism in the skin. Interestingly, immunohistochemical studies already showed that GnRH-, FSH-, and LH receptors are expressed in vessel walls, the epidermis, the hair follicle, and in sebaceous and sweat glands in canine skin (Welle et al., 2006). Moreover, canine hair follicles express sex steroids (Bratka‐Robia et al., 2002), and their cells can metabolize these hormones (Bamberg et al., 2004, 2005). Nevertheless, the role of the skin’s gonadotropin receptors, sex steroid receptors, and metabolism in the pathogenesis or treatment of alopecia X is still unknown.

GnRH analogs are much more potent and sustained in action than endogenous GnRH and have been used extensively in the modulation of sex hormone synthesis in humans (Wu et al., 2021). According to the manufacturer, a 4,7 mg deslorelin implant is designed to be effective for at least 6 months. Our dog showed profuse hair regrowth after 9 months of a single implant, agreeing with another report (Layne & Richmond, 2018) that the efficacy may persist long after the expected medical duration of the implant. As already hypothesized in another study (Albanese et al., 2014) pharmacokinetic variations and individual differences related to the absorption, metabolism, and excretion of deslorelin might help explain why some dogs respond and others do not, as well as why the effect seems to be longer in some patients.

Only one previous study included both neutered females (n=4) and intact males (n=16) when evaluating deslorelin treatment in alopecia X (Albanese et al., 2014). Although 60% of males responded successfully, no hair regrowth was noted in females. This previous study's limitation is the small number of female individuals. Our report breaks this gender paradigm and shows that neutered females with alopecia X can respond to a long-acting GnRH analog.

In the current case, histopathology could not be performed. Still, a previous report in 2 male Keeshonden dogs explored the skin histopathologic findings before and three and a half months after deslorelin treatment. Before treatment, as expected in cases of hair cycle arrest, there was a predominance of kenogen follicles with excessive trichilemmal keratinization, moderate-to-severe follicular hyperkeratosis, and flame follicles. After the implant, anagen follicles predominated, although some follicular units remained in the kenogen phase or telogen, with retained hair shafts surrounded by moderate follicular hyperkeratosis (Layne & Richmond, 2018).

Some of the new hairs in the trunk of our female dog grew with a softer appearance and darker color compared to the original hair. In a previous case report, the same softer appearance but a lighter color was noted after the deslorelin treatment (Layne & Richmond, 2018).

Although some treatment alternatives are currently available for alopecia X, results can vary, as well as costs, risks, and owner adherence. Melatonin is considered the first treatment choice; it can regrow hair in 40-60% of dogs (Frank et al., 2004), but it depends on daily oral administration and owner adherence. The present case tried this drug for 6 months without success. Additionally, trilostane treatment can induce hair regrowth in 85% of Pomeranian dogs (Cerundolo et al., 2004), but costs and side effects can be a concern. On the other hand, deslorelin has shown 60% hair regrowth success when treating alopecia X in 16 male dogs (Albanese et al., 2014) and was considered an uncomplicated, inexpensive, and safe treatment for the present neutered female dog.

## Conclusion

We conclude that a deslorelin implant can be used to treat alopecia X in neutered female dogs. Dogs that fail the treatment with melatonin can respond successfully to a long-acting GnRH analog implant. Profuse hair regrowth can take 9 months after the first implant, but the treatment was considered uncomplicated, cost-effective, and safe. Studies using larger canine cohorts and investigating deslorelin acetate's long-term efficacy and safety in treating alopecia X, regardless of gender, are necessary.

## References

[B001] Albanese F., Malerba E., Abramo F., Miragliotta V., Fracassi F. (2014). Deslorelin for the treatment of hair cycle arrest in intact male dogs. Veterinary Dermatology.

[B002] Amado Martins F., Almeida da Silva G., Ligeiro de Oliveira A. P., Gutierrez Duran C. C., Constantin Baltatu O., Labat Marcos R., Ratto Tempestini Horliana A. C., Regina Zamuner S., Antonio Silva J. (2024). Comparison between melatonin versus melatonin and photobiomodulation versus photobiomodulation in the treatment of Alopecia X in German Spitz dogs: Clinical, randomized, double-blind, parallel, non-inferiority protocol. PLoS One.

[B003] Amaral J., Briantais P., Fontaine C., Rigaut D. (2023). Efficacy and safety of 4.7 mg deslorelin acetate implants as a neutering option in male cats: a large-scale multicentre randomised controlled study. Animals (Basel).

[B004] Bamberg E., Aichinger A., Mitteregger G. (2004). In vitro metabolism of dehydroepiandrosterone and testosterone by canine hair follicle cells. Veterinary Dermatology.

[B005] Bamberg E., Aichinger A., Wünsch G. (2005). In vitro metabolism of progesterone by canine hair follicle cells. Veterinary Dermatology.

[B006] Bratka‐Robia C. B., Egerbacher M., Helmreich M., Mitteregger G., Benesch M., Bamberg E. (2002). Immunohistochemical localization of androgen and oestrogen receptors in canine hair follicles. Veterinary Dermatology.

[B007] Casteel C. O., Singh G. (2020). Physiology, gonadotropin-releasing hormone.

[B008] Cerundolo R., Warren S. (2013). The use of deslorelin to promote hair regrowth in dogs with alopecia X. Veterinary Dermatology.

[B009] Cerundolo R., Lloyd D. H., Persechino A., Evans H., Cauvin A. (2004). Treatment of canine Alopecia X with trilostane. Veterinary Dermatology.

[B010] Cerundolo R., Lloyd D. H., Vaessen M. M., Mol J. A., Kooistra H. S., Rijnberk A. (2007). Alopecia in pomeranians and miniature poodles in association with high urinary corticoid:creatinine ratios and resistance to glucocorticoid feedback. The Veterinary Record.

[B011] Chen C. C., Chuong C. M. (2012). Multi-layered environmental regulation on the homeostasis of stem cells: The saga of hair growth and alopecia. Journal of Dermatological Science.

[B012] Frank L. A. (2005). Growth hormone-responsive alopecia in dogs. Journal of the American Veterinary Medical Association.

[B013] Frank L. A. (2006). Comparative dermatology--canine endocrine dermatoses. Clinics in Dermatology.

[B014] Frank L. A., Hnilica K. A., Oliver J. W. (2004). Adrenal steroid hormone concentrations in dogs with hair cycle arrest (Alopecia X) before and during treatment with melatonin and mitotane. Veterinary Dermatology.

[B015] Frank L. A., Hnilica K. A., Rohrbach B. W., Oliver J. W. (2003). Retrospective evaluation of sex hormones and steroid hormone intermediates in dogs with alopecia. Veterinary Dermatology.

[B016] Gontier A., Youala M., Fontaine C., Raibon E., Fournel S., Briantais P., Rigaut D. (2022). Efficacy and safety of 4.7 mg deslorelin acetate implants in suppressing oestrus cycle in prepubertal female dogs. Animals (Basel).

[B017] Huang H.-P., Lien Y.-H., Chang P.-H. (2009). Effect of castration on hair re-growth in Pomeranians with hair cycle arrest (alopecia X). Journal of Veterinary Clinical Sciences.

[B018] Kang Y. H., Kim M. S., Kang S. Y., Hyun J. E., Hwang C. Y. (2024). Optimal microneedle length for hair regrowth in hair cycle arrest (alopecia X) in six dogs. Veterinary Dermatology.

[B019] Layne E. A., Richmond R. V. (2018). Deslorelin implant treatment for hair cycle arrest (Alopecia X) in two intact male Keeshonden. Journal of the American Animal Hospital Association.

[B020] Lucas X. (2014). Clinical use of deslorelin (GnRH agonist) in companion animals: A review. Reproduction in Domestic Animals.

[B021] Mausberg E. M., Drogemuller C., Rufenacht S., Welle M., Roosje P., Suter M., Leeb T. (2007). Inherited alopecia X in Pomeranians. DTW. Deutsche Tierarztliche Wochenschrift.

[B022] Müntener T., Schuepbach-Regula G., Frank L., Rufenacht S., Welle M. M. (2012). Canine noninflammatory alopecia: A comprehensive evaluation of common and distinguishing histological characteristics. Veterinary Dermatology.

[B023] Stoll S., Dietlin C., Nett-Mettler C. S. (2015). Microneedling as a successful treatment for alopecia X in two Pomeranian siblings. Veterinary Dermatology.

[B024] van Hensbergen I., van den Broek J., van Amersfort K., van der Lee A. (2025). Evaluation of phenotypic risk indicators for the development of alopecia X (hair cycle arrest) in Pomeranian dogs in the Netherlands and Belgium. Veterinary Dermatology.

[B025] Welle M. M. (2023). Canine noninflammatory alopecia: An approach to its classification and a diagnostic aid. Veterinary Pathology.

[B026] Welle M. M., Reichler I. M., Barth A., Forster U., Sattler U., Arnold S. (2006). Immunohistochemical localization and quantitative assessment of GnRH-, FSH-, and LH-receptor mRNA Expression in canine skin: A powerful tool to study the pathogenesis of side effects after spaying. Histochemistry and Cell Biology.

[B027] Wu H. M., Chang H. M., Leung P. C. K. (2021). Gonadotropin-releasing hormone analogs: Mechanisms of action and clinical applications in female reproduction. Frontiers in Neuroendocrinology.

[B028] Zanna G., Abramo F., Contiero B., Zini E., Albanese F., Borio E., Godizzi F., Necci F., Luciani L., Roccabianca P. (2024). Dermoscopic findings and comparison of usefulness of longitudinal versus transversal sections in the histological diagnosis of alopecia X. Veterinary Dermatology.

